# COVID-19 Vaccine Perceptions and Differences by Sex, Age, and Education in 1,367 Community Adults in Ontario

**DOI:** 10.3389/fpubh.2021.719665

**Published:** 2021-09-22

**Authors:** Sabrina K. Syan, Mahmood R. Gohari, Emily E. Levitt, Kyla Belisario, Jessica Gillard, Jane DeJesus, James MacKillop

**Affiliations:** Peter Boris Centre for Addictions Research, McMaster University and St. Joseph's Healthcare Hamilton, Hamilton, ON, Canada

**Keywords:** vaccine, hesitancy, pandemic, COVID-19, attitudes

## Abstract

**Background:** COVID-19 is a global pandemic and vaccination efforts may be impeded by vaccine hesitancy. The present study examined willingness to receive a COVID-19 vaccine, the associated reasons for willingness/unwillingness, and vaccine safety perceptions in a cross-sectional assessment of community adults in Ontario.

**Methods:** One thousand three hundred sixty seven individuals (60.6% female, mean age = 37.5%) participated in this study between January 15, 2021 and February 15, 2021. Perceptions of vaccine safety and reasons for willingness/unwillingness to receive the COVID-19 vaccine were investigated using an online assessment. Perceptions were investigated in general and by age, sex and education using analysis of variance.

**Results:** Overall, 82.8% of the sample reported they were willing to receive a COVID-19 vaccine and 17.2% reported they were unwilling. The three most common reasons for unwillingness were long-term side effects (65.5%), immediate side effects (60.5%), and lack of trust in the vaccine (55.2%). Vaccine willingness significantly differed by sex and education level, with female participants and those with less than a bachelor's degree being more likely to report unwillingness. Perception of COVID-19 vaccine safety was significantly lower (−10.3%) than vaccines in general and differed by age, sex and education, with females, older adults, and individuals with less than a bachelor's degree reporting lower perceived COVID-19 vaccine safety.

**Conclusion:** In this sample of community adults, the COVID-19 vaccine hesitancy rate was less than one in five individuals, but with higher rates in population subgroups. Targeting public health messaging to females and individuals with less than bachelor's degree, and addressing concerns about long-term and immediate side effects may increase vaccine uptake.

## Introduction

The novel coronavirus disease (COVID-19) caused by severe acute respiratory syndrome coronavirus 2 (SARS-CoV-2) was declared a global pandemic by the World Health Organization in March 2020. To date, more than 200 million individuals globally have been infected with COVID-19 and more than 4 million individuals have died (1). Global efforts to mitigate the spread of COVID-19 included travel restrictions, international border closures, and strong public health measures such as physical distancing measures and mask mandates. Complementing these strategies, several vaccines for SARS-CoV-2 have been developed, substantially mitigating the severity of COVID-19 and potentially reducing its transmissibility. In Canada, four vaccines have been approved by Health Canada, three of which are currently being offered to Canadians.

As a mass inoculation program begins, one potential impediment is vaccine hesitancy (i.e., unwillingness to receive the vaccine). A recent systematic review on vaccine hesitancy suggested that willingness to receive the COVID-19 vaccine ranged from 27.7 to 91.3% across the world (2). Further, age, educational status, trust in healthcare and health insurance have all been associated with willingness to receive the COVID-19 vaccine (2–4). The goal of the current study was to clarify the proportion of the population likely to decline a COVID-19 vaccine and the associated reasons. To do so, we examined the prevalence of vaccine willingness/unwillingness and the reasons associated with each perspective in a cross-sectional assessment of general community adults in Ontario. In addition to general perceptions, we examined differences on the basis of sex, age, and education; and perceptions of safety of vaccines in general compared to COVID-19 vaccines. Clarifying reasons for vaccine unwillingness and subgroup differences have the potential to inform public health strategies to address specific concerns, improve vaccine education, and target specific subgroups.

## Method

### Participants and Study Design

This study capitalized on an ongoing longitudinal observational cohort study of health behaviors in community adults. The existing study launched in October 2018 and participants undergo intermittent (biennial or quarterly, depending on the study period) online assessments. Individuals enrolled in the study were ambulatory, non-clinical adults from the Hamilton, Ontario community.

Inclusion criteria included an age between 18 and 65, adequate literacy (at least a ninth-grade education), willingness to consider participation in future research studies, and no extant terminal illness. To enroll in this study, all members of the longitudinal observational cohort study were contacted; the only eligibility criteria to participate were accepting the invitation and completing informed consent. Information related to vaccine hesitancy was collected in the data collection wave from January 15 to February 15, 2021. From the original a longitudinal cohort of 1,502, the current sample comprised 1,367 individuals (91%; Table 1). Demographically, participants were very similar to the local catchment area and similar to provincial and national demographics, albeit with greater representation of females. Comparisons of the sample demographics with municipal, provincial, and national demographics are in Supplementary Materials. The data were collected *via* Research Electronic Data Capture (REDCap) software (5) and participants received a $40 gift card. The study was approved by the Hamilton Integrated Research Ethics Board (Protocol # 4699), all participants underwent informed consent, and all procedures complied with the Helsinki Declaration.

**Table 1:** Participant demographics (*n* = 1,367).

**Demographic variable**	**Mean (SD)/%**
Age	37.5 (14.0)
**Sex**
Male	539 (39.4%)
Female	828 (60.6%)
**Education**
<Bachelor's degree	612 (44.8%)
Bachelor's degree	520 (38.0%)
>Bachelor's degree	235 (17.2%)
**Race**
White	1,078 (78.9%)
Black	20 (1.5%)
Asian	165 (11.9%)
First Nations/Inuit/Metis	13 (1.0%)
Pacific Islander	5 (0.4%)
More than one population group	54 (4.0%)
Other	32 (2.3%)

### Assessment

The assessment is provided in Supplementary Material and comprised purpose-built questions to ascertain willingness to receive the COVID-19 vaccine and examined potential reasons for either receiving or declining the vaccine. These questions were created by study staff and clinicians. Questions regarding the perception of COVID-19 vaccine safety (i.e., “how safe do you believe the COVID-19 vaccines are?”) and general vaccine safety (i.e. “how safe do you believe vaccines are in general?”) were asked using a single item visual analog scale ranging from 0 to 100 with anchors of very unsafe (0) to very safe (100). Individuals that indicated a willingness to receive a COVID-19 vaccine were asked further questions to clarify their reasons for wanting to receive a COVID-19 vaccination. Similarly, individuals that indicated that they were unwilling to receive a COVID-19 vaccination were asked a set of questions to clarify why they were not willing to receive a COVID-19 vaccination. All questions to clarify willingness or unwillingness to receive a COVID-19 vaccine are provided in Supplementary Material. The total number of responses were summed across each question and divided by the total population of willing or unwilling individuals to obtain a percentage.

### Data Analysis

Five quality control questions with unambiguously correct answers (e.g., “in response to this question, please choose option “nearly every day”) were included and participants were excluded for 2+ incorrect responses or if they did not complete the entire survey. Analyses were restricted to unvaccinated individuals. Age and educations subgroups were constructed to map on to commonly used distinctions while approximately balancing sample sizes. Categorical variables were compared across groups using a χ^2^ test. Independent sample T-tests were used to compare continuous variables across two groups. Analyses of variance was used to compare continuous variables across three groups. All statistical analysis was completed using SAS Software and the criterion for statistical significance was *p* < 0.05.

## Results

### Vaccine Willingness

**Figure 1:**
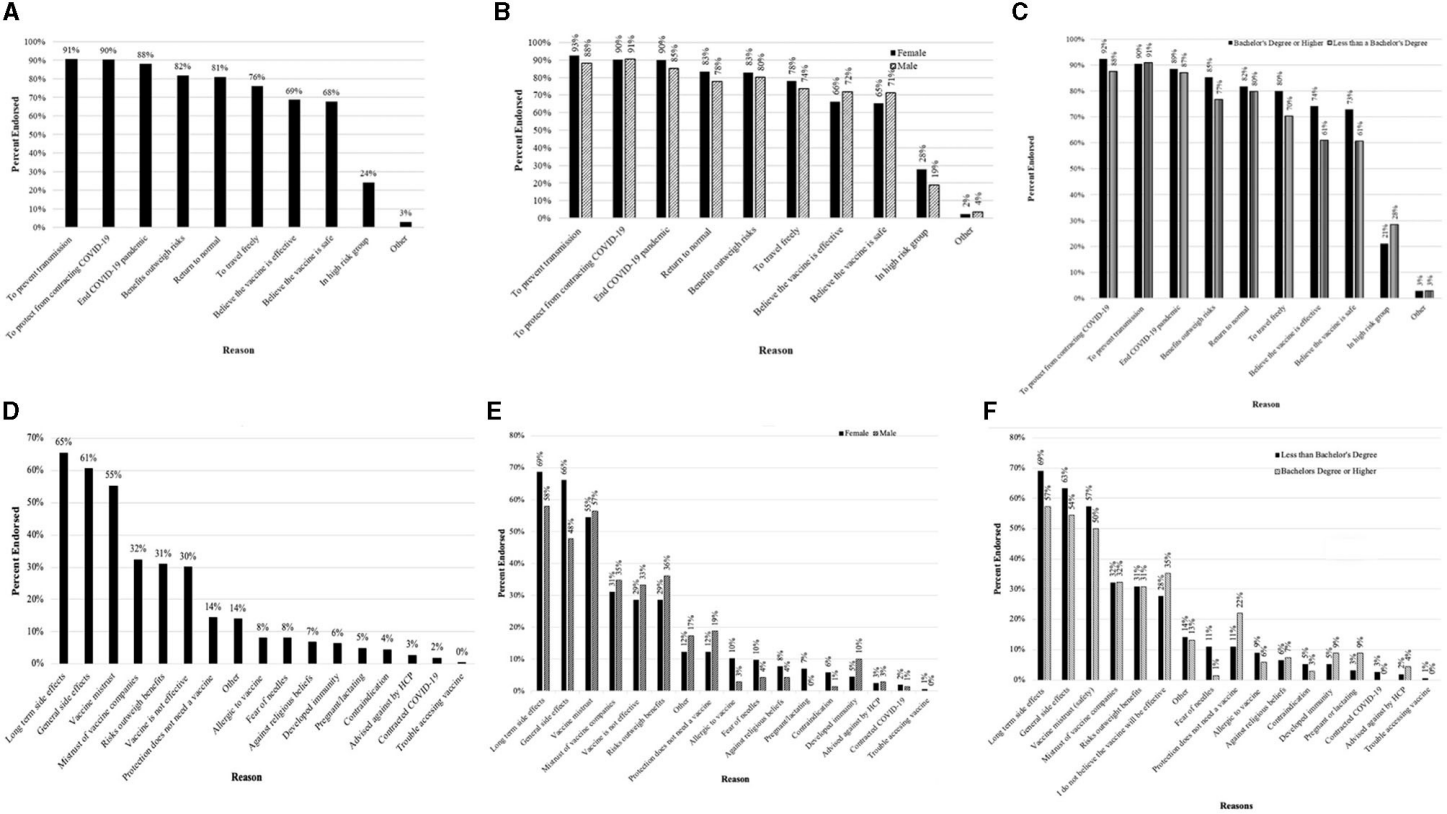
Reasons for receiving the COVID-19 vaccine reported by participants that indicated vaccine willingness **(A–C)** and Reasons for not wanting to receive the COVID-19 vaccine reported by participants that indicated vaccine unwillingness **(D–F)**. **(A)** Overall reasons for receiving the COVID-19 vaccine. **(B)** Reasons for receiving the COVID-19 vaccine reported between male and female participants. **(C)** Reasons for receiving the COVID-19 vaccine reported between participants with less than a Bachelor's degree or a Bachelor's degree or higher. **(D)** Overall reasons for not wanting to receive the COVID-19 vaccine. **(E)** Reasons for not wanting to receive the COVID-19 vaccine across sex. **(F)** Reasons for not wanting to receive the COVID-19 vaccine reported between participants less than a Bachelor's degree or a Bachelor's degree or higher.

Consistent with the vaccine rollout in Canada, only a small subset reported having received at least one dose (5.1%, *n* = 70) at the time of participation and subsequent analyses were restricted to unvaccinated individuals. More than 4 in 5 participants had a favorable perspective on the vaccine (82.8%) (Table 2). Reasons for affirmative willingness are in Figures 1A–C, with prevention of transmission (91%) and protection from contracting COVID-19 (90%) being the most endorsed. For the 17.8% reporting unwillingness, the reasons for unwillingness are in Figures 1D–F, with long-term side effects (65.5%), immediate side effects (60.5%), and a lack of trust in the vaccine itself (55.2%) being the three most common reasons for not wanting to receive the vaccination.

**Table 2:** COVID-19 vaccination willingness in general and by subgroup among unvaccinated participants (*n* = 1297).

**Demographic Subgroup**	**Unwilling (No)**	**Willing (yes)**	**χ^2^**	***P***
Total sample	223 (17.2%)	1,074 (82.8%)		
Sex			8.93	0.003
Male	70 (13.4%)	453 (86.6%)		
Female	153 (19.8%)	621 (80.2%)		
Age (years)			5.67	0.059
< 30	84 (14.8%)	482 (85.2%)		
30–49	86 (20.6%)	331 (79.4%)		
50+	53 (16.9%)	261 (83.1%)		
Education			63.70	<0.001
< Bachelor's degree	155 (25.9%)	443 (74.1%)		
Bachelor's degree	57 (11.7%)	430 (88.3%)		
>Bachelor's degree	11 (5.2%)	201 (94.8%)		

*The frequencies above were calculated by χ^2^ between the percent of individuals that indicated that they were willing to receive the vaccination (Yes) or unwilling to receive the vaccination (No)*.

Willingness to receive the COVID-19 vaccine differed by sex and education, but not age (Table 2). Males reported modestly higher willingness than females (86.6 vs. 80.2%). With regards to education, individuals with lower than a bachelor's degree were also more likely to refuse a COVID-19 vaccination; 25.9% of individuals with less than a bachelor's degree education indicated that they were not willing to receive a COVID-19 vaccine, as compared to 11.7% with a bachelor's degree level education and 5.2% with at least a graduate level education (Table 2).

Differences in reasons for willingness by sex and education are in Figure 1. Female participants and those with higher education more commonly endorsed wanting to prevent transmission and protection from contracting COVID-19. Differences in reasons for unwillingness by sex and education are in Figures 1D–F. In this case, female participants and those with less than a bachelor's degree reported that they worried about long term and immediate vaccine side effects than male participants and participants with a bachelor's degree or higher.

### Vaccine Safety

With regards to vaccine safety, participants endorsed significantly greater perception of general vaccine safety (84.5) as compared to COVID-19 vaccine safety (74.2). Given the scaling, this reflects an average perception of 10.3% lower safety.

Subgroup differences were present based on sex, age, and education (Table 3). Male participants endorsed higher vaccine safety perception for general vaccines and the COVID-19 vaccines, compared to female participants. Individuals under 30 years of age endorsed a significantly higher perception of both general and COVID-19 vaccine safety as compared to individuals in the oldest age bracket, over 50 years of age (*p* < 0.05). General or COVID-19 vaccine safety perceptions did not differ significantly between the under 30- and 30–49-year age brackets. Individuals with less than a bachelor's degree indicated lower perception of general and COVID-19 vaccine safety than both higher education groups (*p*s < 0.05) and individuals with greater than a bachelor's degree (*p* < 0.05). There was not a significant difference in perception of general or COVID-19 vaccination safety between individuals with a bachelor's degree and those with greater than a bachelor's degree.

**Table 3:** Perception of general and COVID-19 vaccine safety among participants (*n* = 1,367). Note that COVID-19 vaccines were not differentiated by specific product.

	**General vaccine safety perception (0–100)**		**COVID-19 vaccine safety perception (0–100)**	
	**Mean (SEM)**	***P***	**Mean (SEM)**	***P***
Total	84.5 (0.51)		74.2 (0.67)	
Sex		<0.001		0.002
Male	86.6 (0.74)		76.7 (1.03)	
Female	83.2 (0.68)		72.6 (0.87)	
Age (years)		<0.001		0.015
<30	87.1 (0.68)		77.0 (0.92)	
30–49	83.8 (0.97)		71.3 (1.28)	
50+	81.6 (1.12)		72.9 (1.39)	
Education		<0.001		<0.001
< Bachelor's degree	80.4 (0.87)		67.3 (1.12)	
Bachelor's degree	87.2 (0.71)		78.8 (0.92)	
> Bachelor's degree	89.4 (0.86)		82.2 (1.22)	

## Discussion

This study evaluated willingness to receive the COVID-19 vaccination and perceptions of vaccine safety of the COVID-19 vaccination in a community-based sample of adults from Southern Ontario. Results indicate that although a large proportion of the sample was willing to receive the vaccination, more than 4 in 5, a sizable fraction of the population (17.2%) was not. Individuals who are not willing to receive the vaccination indicated that concerns regarding immediate and long-term vaccine side effects and a lack of trust in the vaccine itself are their main reasons for not wanting to receive the vaccination. Vaccine willingness increased with level of education and significantly differed between male and female participants, with female participants reporting that they are less willing to be vaccinated.

Participants reported that they considered the COVID-19 vaccine 10.3 less safe than they viewed general vaccine safety. This may be due to the speed with which the COVID-19 vaccination was created, and expedited approval times associated with the vaccinations as compared to general vaccination development process which may take a decade or longer. Furthermore, the evolving narrative surrounding information regarding COVID-19, COVID-19 variants and the efficacy of vaccinations against potential variants may also be a contributing factor to vaccine hesitancy. Specific to female participants, concerns regarding vaccinations and fertility may play a role in making female participants less willing to receive the COVID-19 vaccine and may suggest that they believe it is less safe. It should be noted that 7% of women reported that their unwillingness to receive the vaccination was due to pregnancy or lactation. Recent evidence regarding the safety and efficacy of the COVID-19 vaccinations in pregnant and lactating women and conferred benefits to newborns as a result are emerging (6), but would not have been available at the time of the assessment. Our results are consistent with other recently published studies. A study investigating vaccine willingness in the United States found that females were less willing to receive the COVID-19 vaccine than males (3). Lower willingness among females as compared to males was also noted in a study investigating COVID-19 vaccine willingness in Israel (7).

Perceptions of general and COVID-19 vaccine safety both decreased with age and increased with education. Perceptions of vaccine safety (but not willingness) differed by age; although older adults (50 years of age or older) reported that they were still willing to receive the vaccination. Vaccine willingness did not differ by age, however, did differ by education—with individuals with less than a Bachelor's degree indicating that they were 13.1% less willing as individuals with greater than a Bachelor's degree to receive the vaccination. This is consistent with recent findings from the United States that individuals with higher education are significantly more likely to get a COVID-19 vaccination and to believe in the effectiveness and safety of the vaccine (8). Recently published studies investigating COVID-19 vaccine willingness in various countries around the world have found that low education is associated with decreased willingness to receive the COVID-19 vaccine (3, 7, 9, 10). Education is also associated with greater engagement in pro-health behaviors (11, 12) such as vaccinations and lower delay discounting (13), a measure of impulsivity that refers to the decline in value of a reward based on the delay to its receipt that has been linked to numerous health behaviors, including vaccine uptake (14, 15).

The results of this manuscript highlight several implications for public health messaging to maximize vaccine uptake. The most common concerns highlighted by individuals in our study that were unwilling to receive the vaccine included concerns regarding long term and more general vaccine side effects and general vaccine mistrust. Effective public health messaging to combat vaccine hesitancy should focus on providing information regarding the immediate and long-term vaccine side effects in an effort to improve vaccine willingness and perceptions of vaccine safety. Further, results highlight that female participants and those with less than a bachelor's level education endorsed greater concerns with regards to long-term and general vaccine willingness as compared to male or more educated participants and may need to be the focus of enhanced public health messaging. Targeting public health messaging to these subpopulations may increase vaccine willingness and update not only for first, but also second doses of vaccinations.

This study should be considered in the context of its strengths and limitations. Amongst its strengths, it systematically assessed vaccine willingness, reasons, and safety, and provides timely information that may guide public health efforts to decrease hesitancy and increase vaccine uptake. The sample size was relatively large which allowed for high statistical power and the sample was relatively representative of community adults in Canada (see Supplementary Materials), albeit with a higher rate of females and somewhat lower racial diversity, although that reflects the local catchment area. The relatively low racial diversity is a limitation, so we are not able to comment on willingness to receive the COVID-19 vaccination or safety perceptions within specific racial groups. Furthermore, the results may be less generalizable to highly racially diverse catchment areas. Similarly, the sample was recruited from an urban/suburban catchment area and thus lacks rural representation. An additional limitation of this study is that the online assessment and questions asked provided a snapshot of health attitudes which may not always translate to health behavior.

Taken together, in this large community-based sample, the large majority of individuals reported being willing to receive the COVID-19 vaccination, but a non-trivial fraction was not, principally due to concerns regarding vaccine side effects and a lack of trust in the vaccine itself. This suggests that public health messaging to combat vaccine hesitancy should focus on the safety profile of the approved COVID-19 vaccinations and consider targeting the segments of the population reporting the greatest unwillingness to vaccination.

## Data Availability Statement

The data will be made available in response to reasonable and appropriate requests based on approval of the corresponding author and the institutional ethics board.

## Ethics Statement

All procedures were reviewed and approved by Hamilton Integrated Research Ethics Board. All participants underwent informed consent to participate in this study.

## Author Contributions

JM, SS, MG, EL, and KB were responsible for study design. JG and JD were responsible for study coordination and data collection. MG and KB were responsible for data analysis. SS and JM drafted the manuscript. All authors edited and approved the final version of the manuscript.

## Funding

This research was supported by the Canadian Institutes of Health Research and the Peter Boris Chair in Addictions Research.

## Conflict of Interest

JM is a principal and senior scientist in BEAM Diagnostics, Inc. and a consultant to Clairvoyant Therapeutics, Inc., but no related products or services were used in this research. The remaining authors declare that the research was conducted in the absence of any commercial or financial relationships that could be construed as a potential conflict of interest.

## Publisher's Note

All claims expressed in this article are solely those of the authors and do not necessarily represent those of their affiliated organizations, or those of the publisher, the editors and the reviewers. Any product that may be evaluated in this article, or claim that may be made by its manufacturer, is not guaranteed or endorsed by the publisher.
