# Supplementary material for: COVID-19 Vaccine Perceptions and Differences by Sex, Age, and Education in 1,367 Community Adults in Ontario
Source: Front Public Health. 2021 Sep 22;9:719665. doi: 10.3389/fpubh.2021.719665 (PMC8494003; doi:10.3389/fpubh.2021.719665)
Supplement: Supplementary file 1 [file Table_1.docx]

**SUPPLEMENTAL MATERIALS**

Table S1: Assessment question to ascertain vaccine willingness

| Selection/Option | Question |
| --- | --- |
| 2 | Yes, I have already taken the vaccine |
| 1 | Yes, I will take the vaccine |
| 0 | No, I will not take the vaccine |

Table S2: Questions to clarify reasons for vaccine willingness (affirmative responses)

| Item | | Question |
| --- | --- | --- |
| 1 | To protect myself from contracting COVID-19 | |
| 2 | To prevent transmission to my family, friends, or other contacts | |
| 3 | I am in a high-risk group (e.g., elderly, immunocompromised, essential worker, etc.) | |
| 4 | I believe the vaccine is safe | |
| 5 | I believe the vaccine is effective | |
| 6 | I believe the benefits outweigh the risks | |
| 7 | To return to “normal” activities (e.g., no masks, no distancing, etc.) | |
| 8 | To travel more freely | |
| 9 | To help end the COVID-19 pandemic | |
| 10 | Other | |

Table S3: Questions to clarify reasons for vaccine unwillingness (negative responses)

| Item | | Question |
| --- | --- | --- |
| 1 | I have been advised by a healthcare provider not to get the vaccine | |
| 2 | I have allergies or a history of allergies with vaccines | |
| 3 | I have a previous symptom or condition that makes treatment risky (e.g., vaccine contraindication) | |
| 4 | I am pregnant or lactating | |
| 5 | I have a fear of needles | |
| 6 | I do not trust that the vaccine is safe | |
| 7 | I am worried about the side effects of the vaccine | |
| 8 | I am worried about the long-term effects of the vaccine | |
| 9 | I do not believe the vaccine will be effective | |
| 10 | I believe the risks outweigh the benefits | |
| 11 | It goes against my personal or religious beliefs (e.g., Contains objectionable ingredients, not kosher/halal/vegan/cruelty free) | |
| 12 | I do not trust the companies and/or governments providing the vaccine | |
| 13 | I have or will have trouble accessing the vaccine (e.g., mobility issues or travel issues) | |
| 14 | I have already contracted COVID-19 | |
| 15 | I believe I have already developed immunity | |
| 16 | I believe protection against COVID-19 does not require a vaccine | |
| 17 | Other | |

Table S4: Assessment questions that used a visual analogue scale

| Question | Visual Analog Scale: 0 (Very Unsafe) – 100 (Very Safe) |
| --- | --- |
| Please rate how safe you believe vaccines are in general | 0-100 |
| Please rate how safe you believe the COVID-19 vaccines are | 0-100 |

Table S2. Demographics for the original longitudinal study cohort (*n* = 1502), the local catchment area (Hamilton, ON), province, and nation. Municipal, provincial, and national demographics are from the Statistics Canada 2016 census data.

|  | Study Cohort | Hamilton | Ontario | Canada |
| --- | --- | --- | --- | --- |
| Sex (% male) | 39.7% | 48.7% | 48.8% | 49.1% |
| Age (mean) | 37.5 | 41.6 | 41.0 | 41.2 |
| Race (% non-visible minority) | 80.5% | 82% | 71% | 77.7% |
| Education (median) | Bachelor’s | College; CEGEP or other non-university certificate or diploma | College; CEGEP or other non-university certificate or diploma | College; CEGEP or other non-university certificate or diploma |
| Household Income (median) | $60,000-74,999 | $70,000- 79,999 | $70,000- 79,999 | $45,000-59,999 |
